# *Trichoderma*-Induced Resistance to *Botrytis cinerea* in *Solanum* Species: A Meta-Analysis

**DOI:** 10.3390/plants11020180

**Published:** 2022-01-11

**Authors:** Samuele Risoli, Lorenzo Cotrozzi, Sabrina Sarrocco, Maria Nuzzaci, Elisa Pellegrini, Antonella Vitti

**Affiliations:** 1 University School for Advanced Studies IUSS Pavia, Piazza della Vittoria 15, 27100 Pavia, Italy; samuele.risoli@iusspavia.it; 2 Department of Agriculture, Food and Environment, University of Pisa, Via del Borghetto 80, 56124 Pisa, Italy; elisa.pellegrini@unipi.it; 3 Nutrafood Research Center, University of Pisa, Via del Borghetto 80, 56124 Pisa, Italy; 4 School of Agricultural, Forestry, Food and Environmental Sciences, University of Basilicata, Viale dell’Ateneo Lucano 10, 85100 Potenza, Italy; maria.nuzzaci@unibas.it (M.N.); avitti@unisa.it (A.V.); 5 Department of Pharmacy, University of Salerno, Via Giovanni Paolo II, 132, 84084 Fisciano, Italy

**Keywords:** grey mold, systemic resistance, pathogenesis-related (PR) genes, defense-signal transduction pathway, *Trichoderma* spp.

## Abstract

With the idea of summarizing the outcomes of studies focusing on the resistance induced by *Trichoderma* spp. against *Botrytis cinerea* in tomato, the present paper shows, for the first time, results of a meta-analysis performed on studies published from 2010 to 2021 concerning the cross-talk occurring in the tomato–*Trichoderma*-*B. cinerea* system. Starting from an initial set of 40 papers, the analysis was performed on 15 works and included nine parameters, as a result of a stringent selection mainly based on the availability of more than one article including the same indicator. The resulting work not only emphasizes the beneficial effects of *Trichoderma* in the control of grey mold in tomato leaves (reduction in disease intensity, severity and incidence and modulation of resistance genes in the host), but carefully drives the readers to reply to two questions: (i) What are the overall effects of *Trichoderma* on *B. cinerea* infection in tomato? (ii) Do the main effects of *Trichoderma* differ based on the tomato species, *Trichoderma* species, amount, type and duration of treatment? At the same time, this meta-analysis highlights some weak points of the available literature and should be seen as an invitation to improve future works to better the conceptualization and measure.

## 1. Introduction

Tomato (*Solanum lycopersicum*) is one of the most cultivated horticultural crops worldwide, with more than 180 million tons produced over an area of about 5 million hectares in 2019 [1]. Tomato is also the most consumed vegetable worldwide and a major component of the Mediterranean diet, mainly because of its remarkable nutraceutical properties due to the considerable presence of antioxidant compounds, such as lycopene, ascorbic acid, phenols, flavonoids and tocopherols [2]. At the same time, tomato is one of the most economically important host plants of *Botrytis cinerea* (a pathogenic fungus belonging to the phylum Ascomycota causing grey mold [3]). 

*Botrytis cinerea* has been classified as the second most dangerous plant pathogen [4] because of its wide host range (including more than 200 species over both temperate and tropical areas) and its ability to attack leaves, stems, flowers and fruits, so determining severe pre- and post-harvest losses [5,6], as well as the low efficacy of chemical control due to its wide genetic variability and high capacity to acquire resistance against chemical fungicides [7]. For these reasons, *B. cinerea* has received increasing attention, also becoming a model pathogen in several studies focused on its management [6]. Specifically, a strong effort has been made to develop complementary, or rather alternative control strategies to agrochemicals, also because of the well-known harmful effects of such products on both environmental and human health [8]. In this context, special attention has been paid to tomato protection, mainly because tomato cultivars with adequate resistance to *B. cinerea* infection are not available yet [3]. 

A complementary/alternative method to agrochemicals with a great potential to protect plants from *B. cinerea* infection is represented by the biological control through the use of beneficial fungi belonging to the *Trichoderma* genus [8]. *Trichoderma* spp. act as biocontrol agents against the pathogen both directly, through a series of mechanisms including mycoparasitism, competition for nutrients and space, fungistasis, antibiosis and/or modification of the rhizosphere [9], and indirectly, by the induction of host plant defense as systemic acquired resistance (SAR) and induced systemic resistance (ISR) [10,11]. This indirect mode of action is mediated by the regulation of a complex network of host plant signal transduction pathways. It involves several molecules and hormones, among which reactive oxygen species (ROS), salicylic acid (SA), jasmonic acid (JA) and ethylene (Et), and the cross-talk between them assume considerable relevance; meanwhile, pathogenesis-related (PR) genes are a series of marker genes responsible for the activation of SA, JA, and Et signaling and, therefore, are involved in these defense-signal transduction pathways [12,13,14,15]. In particular, as reviewed by Vos et al. [8], the resistance to *B. cinerea* in tomato has been demonstrated to be mainly dependent on complex, often overlapping, SA- and JA/Et-mediated signal transduction pathways, a claim already supported by Harman et al. [10].

The first attempt to investigate the effectiveness of the biological control of *B. cinerea* infection by using *Trichoderma* spp. in tomato dates back to the 1990s [16]; in those years, an induction of systemic resistance against tomato grey mold was firstly and precisely attributed to *T. harzianum* [17]. Since then, several classes of *T. harzianum*-derived metabolites have been indicated to be able of inducing specific tomato defense responses against *B. cinerea* [18]. Despite a number of studies have been carried out to understand the mechanisms involved in this kind of plant-mediated defense responses by *Trichoderma* spp., a general understanding of how those responses can vary depending on specific interactions between tomato and *Trichoderma* species and/or treatment is still lacking. Meta-analytic techniques may provide an objective means to quantitatively summarize the data provided by available studies focused on the *Trichoderma*–tomato-*B. cinerea* interaction. To the best of our knowledge, however, no study has explored this approach, a gap we address in this research.

The objective of this meta-analytic paper was to summarize and synthesize the outcomes of studies published from 2010 to 2021 on the resistance induced by *Trichoderma* spp. against *B. cinerea* in tomato. The idea to fix 2010 as time zero to collect papers to be submitted to meta-analysis originally arose from the publication by Shoresh et al. [19], a review on ISR and plant responses to fungal biocontrol agents, where the ability of *Trichoderma* spp. to reprogram plant gene expression was deeply described. In the same volume, a contribution by Lorito et al. [20], focusing on translational research on *Trichoderma*, starting from “omics” to move to the field, was released. Taking into account these two papers and considering the increasing of data referring to the cross-talk in the tri-partite plant–pathogen–biocontrol agent interaction due to the use of Next Generation Sequencing (NGS) technologies in plant pathology from 2008 [21], it was our opinion that 2010–2021 could have been a reasonable timeframe to analyze an exhaustive collection of research papers on the topic here proposed.

The following questions were specifically addressed: (i) What are the overall effects of *Trichoderma* on *B. cinerea* infection in tomato? (ii) Do the effects of *Trichoderma* differ based on the *Solanum* species, *Trichoderma* species, amount, type and duration of treatment? 

## 2. Results

### 2.1. Overview on the Database of the Effects of Trichoderma on Tomato/B. cinerea Interaction

Starting from 25 parameters originally recorded from the reviewed studies, the nine parameters included in the present meta-analysis after the application of exclusion criteria (i.e., those which recorded a minimum of six observations or originated from at least two independent papers) regarded *B. cinerea* infection (i.e., disease intensity, severity and incidence) and defense-related genes involved in JA (i.e., proteinase inhibitors I and II, *PINI* and *PINII*, and tomato lipoxygenase A and C, *TomloxA* and *TomloxC*) and SA (*PR1b1* and *PR-P2*) pathways in tomato (see Section 4.1 and Section 4.3 for further details about classification of *B. cinerea* infection parameters and parameter exclusion criteria, respectively). Discarded parameters included other defense-related genes involved in JA (i.e., multicystatin, *MC*; peptide prosystemin, *PS*; tomato lypoxigenase D, *TomloxD*), SA (i.e., β-1,3-Glucanase, *GluB*; *osmotin*; *PR1a*; SA methyltransferase, *SAMT*) and Et pathways (i.e., methionine synthase, *MS*; DNA-binding protein Pti4, *Pti4*; pathogenesis-related genes transcriptional activator, *Pti5*), as well as leaf JA, SA and abscisic acid (ABA) contents. 

The 15 studies selected for the present meta-analysis (Appendix A) investigated six tomato species: *S. lycopersicum* (the only cultivated species) was explored by all studies, whereas *S. habrochaites* was examined only by two works, and *S. chilense*, *S. lycopersicoides*, *S. pennellii*, and *S. pimpinelli-folium* were investigated only by one study. Sixty percent of the studies performed the *B. cinerea* inoculation on mature plants, whereas the remaining 40% of the works inoculated seedlings. The concentrations of *B. cinerea* suspensions used for inoculations ranged from 5.0 × 10^4^ to 2.0 × 10^8^ spores mL^−1^, with an average value of 1.55 × 10^6^ spores mL^−1^ (applied by spray or spot inoculation). Eight *Trichoderma* species were investigated: *T. harzianum* in seven studies, *T. asperellum* in three works, *T. atroviride* and *T. virens* in two studies, and *T. arundinaceum T. koningiopsis*, *T. longibrachiatum* and *T. parareseei* only in one study. Most of the studies (*n* = 9) applied *Trichoderma* to the soil, whereas four works performed a seed treatment, and only two studies conducted a leaf application. *Trichoderma* treatments used suspension concentrations ranging from 1.0 × 10^4^ to 2.0 × 10^8^ spores mL^−1^, with an average value of 2.13 × 10^7^ spores mL^−1^. Sixty percent of the studies applied *Trichoderma* at the seedling stage, whereas four studies did so at sowing, and only in two works were mature plants treated. *Trichoderma* treatments lasted 0–5, 6–20, 21–35, and >35 days in three, six, seven and two studies, respectively. Analyses were performed at 0–3, 4–7, 8–14 and >14 days of *B. cinerea* infection in four, ten, five and one studies, respectively.

### 2.2. Overall Effects of Trichoderma 

**Figure 1:**
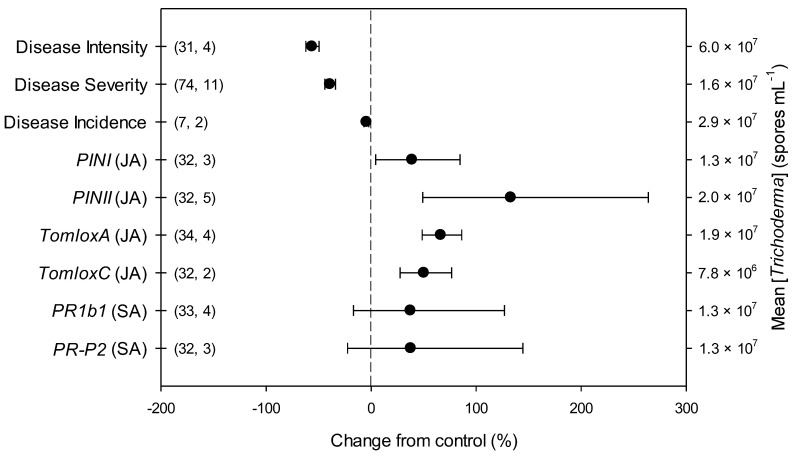
Effects of *Trichoderma* against *Botrytis cinerea* infection (i.e., disease intensity, severity and incidence) and defense-related genes involved in jasmonic (JA; i.e., proteinase inhibitors I and II, *PINI* and *PINII*, and tomato lipoxygenase A and C, *TomloxA* and *TomloxC*) and salicylic acid (SA; pathogenesis-related 1b1 and P2, *PR1b1* and *PR-P2*) pathways in tomato leaves. Symbols represent the mean percentage change due to *Trichoderma* treatment relative to control (i.e., *B. cinerea* infection without *Trichoderma* treatment), and the bars show the 95% confidence interval. Number of observations and papers are shown in parentheses; mean *Trichoderma* spp. concentrations are given along the right *y* axis.

The overall effects of *Trichoderma* on parameters related to *B. cinerea* infection and defense-related genes in tomato included in the present meta-analysis (*n* = 9) are shown in Figure 1 and Table S2. Across all studies, disease intensity, severity and incidence respectively decreased by 56, 39 and 5% due to *Trichoderma* treatment, in comparison with untreated controls. *Trichoderma* also induced an overexpression of all the investigated defense-related genes involved in JA pathway, i.e., *PINI*, *PINII*, *TomloxA* and *TomloxC* (+39, +133, +66 and +50%, respectively). Differently, no *Trichoderma*-induced changes were observed for the investigated defense-related genes involved in SA pathway, i.e., *PR1b1* and *PR-P2*.

### 2.3. Differences in Effects of Trichoderma within Descriptive Categories

**Figure 2:**
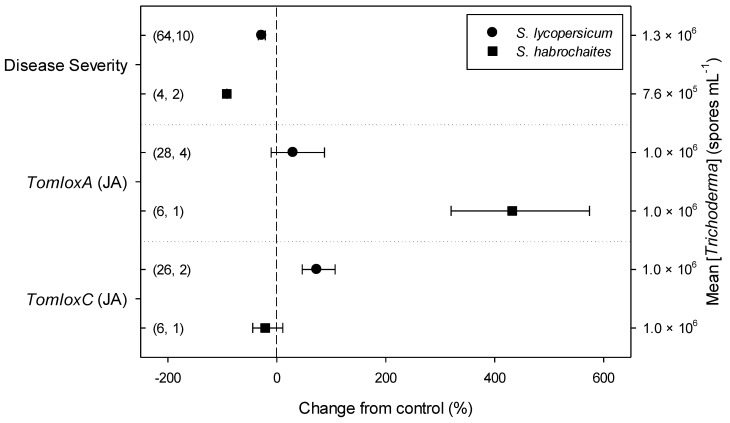
Differential effects of *Trichoderma* on *Botrytis cinerea* infection (i.e., disease severity) and defense-related genes involved in jasmonic acid (JA; i.e., tomato lipoxygenase A and C, *TomloxA* and *TomloxC*) pathway in tomato leaves on the basis of tomato species (*Solanum lycopersicum* and *S. habrochaites*). Symbols represent the mean percentage change due to *Trichoderma* treatment relative to control (i.e., *B. cinerea* infection without *Trichoderma* treatment), and the bars show the 95% confidence interval. Number of observations and papers are shown in parentheses; mean *Trichoderma* spp. concentrations are given along the right *y* axis.

**Figure 3:**
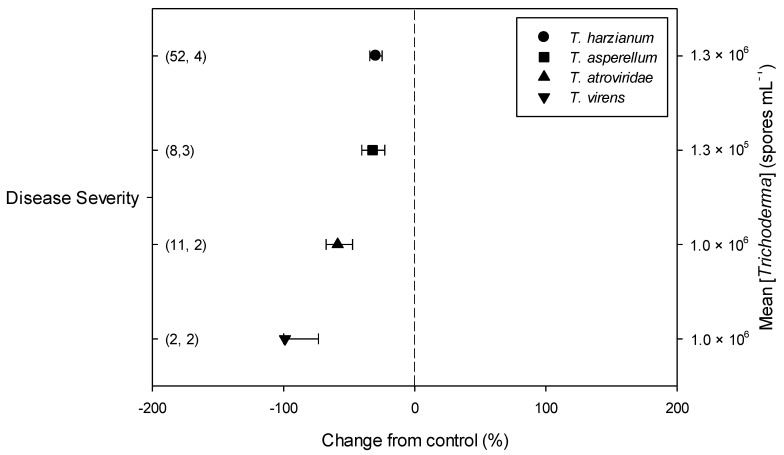
Differential effects of *Trichoderma* on *Botrytis cinerea* infection (i.e., disease severity) in tomato leaves on the basis of *Trichoderma* species (*Trichoderma harzianum*, *T. asperellum*, *T. atroviridae* and *T. virens*). Symbols represent the mean percentage change due to *Trichoderma* treatment relative to control (i.e., *B. cinerea* infection without *Trichoderma* treatment), and the bars show the 95% confidence interval. Number of observations and papers are shown in parentheses; mean *Trichoderma* spp. concentrations are given along the right *y* axis.

**Figure 4:**
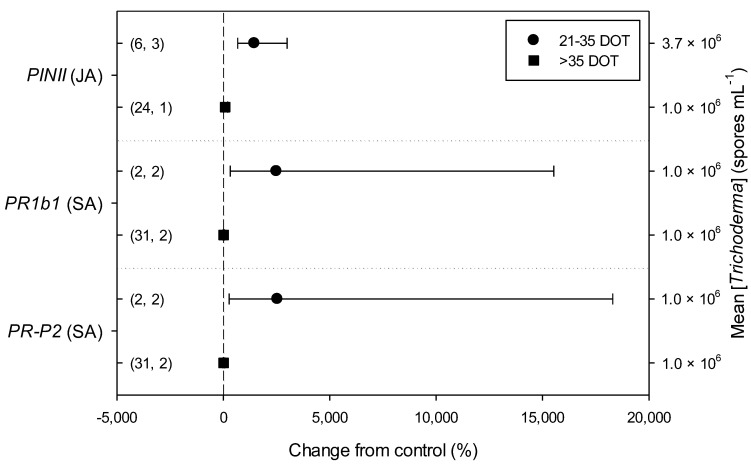
Differential effects of *Trichoderma* on defense-related genes involved in jasmonic (JA; i.e., proteinase inhibitors II, *PINII*) and salicylic acid (SA; pathogenesis-related 1b1 and P2, *PR1b1* and *PR-P2*) pathways in tomato leaves infected by *Botrytis cinerea* on the basis of duration of *Trichoderma* treatment (21–35 and >35 days of treatment, DOT). Symbols represent the mean percentage change due to *Trichoderma* treatment relative to control (i.e., *B. cinerea* infection without *Trichoderma* treatment), and the bars show the 95% confidence interval. Number of observations and papers are shown in parentheses; mean *Trichoderma* spp. concentrations are given along the right *y* axis.

**Figure 5:**
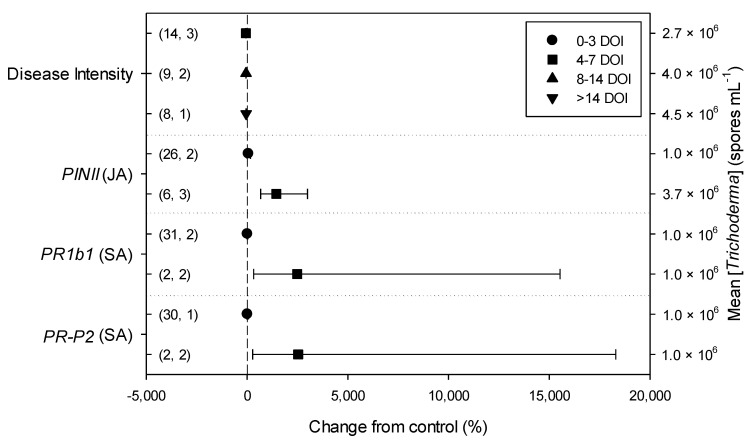
Differential effects of *Trichoderma* on *Botrytis cinerea* infection (i.e., disease intensity) and defense-related genes involved in jasmonic (JA; i.e., proteinase inhibitors II, *PINII*) and salicylic acid (SA; pathogenesis-related 1b1 and P2, *PR1b1* and *PR-P2*) pathways in tomato leaves on the basis of duration of *B. cinerea* infection (0–3, 4–7, 8–14 and >14 days of infection, DOI). Symbols represent the mean percentage change due to *Trichoderma* treatment relative to control (i.e., *B. cinerea* infection without *Trichoderma* treatment), and the bars show the 95% confidence interval. Number of observations and papers are shown in parentheses; mean *Trichoderma* spp. concentrations are given along the right *y* axis.

Meta-regression outcomes for descriptive categories are shown in Table 1 and Table S3 (numbers of observations and studies adopted for inclusion/exclusion of parameters and levels of descriptive categories in the meta-regression are reported in Tables S4–S10). To note, disease incidence was never analyzable by meta-regression after exclusion criteria were assessed. Among the reported tomato species, only *S. lycopersicum* and *S. habrochaites* were comparable (Figure 2, Table S4): disease severity decreased more in *S. habrochaites* than in *S. lycopersicum* (−92 and −28%, respectively); *TomloxA* resulted overexpressed only in *S. habrochaites* (+432%), whereas *TomloxC* only in *S. lycopersicum* (+73%); no difference in the effects of *Trichoderma* was reported for the expression of *PINI*, *PR1b1* and *PR-P2*. The tomato phenological stage at *B. cinerea* (Table S5) infection and at *Trichoderma* (Table S8) treatment did not affect the effect of *Trichoderma* application on disease severity, the only parameter comparable after exclusion criteria were assessed. Differently, disease severity decreased more when *T. virens* was applied (−99%) than when the treatment was performed with *T. atroviridae* (−59%), and even more than when *T. harzianum* and *T. asperellum* were used (−30 and −32%, respectively; Figure 3; Table S6). No differences in the effects of *Trichoderma* on all the investigated defense-related genes were reported on the basis of *Trichoderma* species (only *T. harzianum* and *T. atroviridae* were comparable). *Trichoderma* treatment types (only seed and soil applications were comparable, Table S7) did not affect the effects of *Trichoderma* on disease severity and *PINII* expression. Finally, the duration of *Trichoderma* treatment (Table S9) did not affect its effect on disease severity (6–20 vs. 21–35 vs. >35 day of treatment, DOT), *PINI* and *TomloxA* (21–35 vs. >35 DOT); conversely, *PINII* was more overexpressed for *Trichoderma* treatments lasting from 21 to 35 days than for longer treatments (i.e., >35 DOT; +1447 and 21%, respectively), and *PR1b1* and *PR-P2* were overexpressed for 21–35 DOT (around +2500%), but not for >35 DOT (Figure 4). Finally, the reduction in disease intensity due to *Trichoderma* treatment was more pronounced at 4–7 days of *B. cinerea* infection than at 8–14 or >14 days of infection (DOI), *PINII* was more overexpressed at 4–7 DOI than at 0–3 DOI (+1447 and +64%, respectively), and *PR1b1* and *PR-P2* were overexpressed at 4–7 DOI (around +2500%), but not at 0–3 DOI (Figure 5). No differential effects of *Trichoderma* treatment on disease severity (0–3 vs. 4–7 vs. 8–14 DOI), *PINI* and *TomloxA* (0–3 vs. 4–7 DOI) were reported on the basis of duration of *B. cinerea* infection (Table S10).

## 3. Discussion

From 1932, when Weindling published the first paper describing the beneficial effects of *Trichoderma* sp. [22] in terms of plant protection from pathogens, a huge amount of research has been performed—focusing on these fungi as biocontrol agents in different pathosystems—to the point that *Trichoderma* spp. actually represent one of the most used beneficial organisms developed as active ingredients of commercial biopesticides [23]. Despite *Trichoderma* spp. being able to exploit several mechanisms of action to protect plants from biotic stresses, the modulation of the defense response in the host is the one that has received the most attention in recent decades [19], which is also supported by the availability of modern NGS technologies that allow a deeper investigation of the molecular mechanisms underpinning the dialogue occurring among the biocontrol agent, pathogen and plant [24]. With the aim of providing an overview of the most recent available literature, as far as we know, the present work reports, for the first time, results obtained from a meta-analysis of papers concerning the *Trichoderma*–tomato–*Botrytis cinerea* interaction, focusing on the induction of resistance as a result of the cross-talk occurring in this tritrophic system.

From an initial pool of 40 papers published in the timeframe 2010–2021, the analysis was performed on 9 out of 25 parameters originally included in the 15 selected works (Table S1 and Appendix A), thus apparently showing a limited number of observations, if compared with other meta-analysis whose topics are about the effect of “good” fungi on host plants [25,26], but almost in line with others where the same range (seven indicators) was used [27]. The choice of the parameters to be excluded from the meta-analysis was driven by the availability of more than one article including them. This is the case of defense-related genes such as those belonging to the Et pathway, as well as quantification of JA, SA and ABA, thus limiting the possibility to furnish to the reader with a wider overview of host response in the tri-partite system. In any case, the genes selected here (as well as the parameters used to evaluate the disease level) are those included among several authors to determine if and how the plant defense response is activated with the involvement of both the SA- and JA/Et-mediated signal transduction pathways [8]. 

On the whole, the analysis reported here emphasizes the positive effect of *Trichoderma* spp. in the control of grey mold in tomato leaves, both as a significant reduction in disease intensity, severity and incidence, as well as an overexpression of some genes, connected with resistance, such as those involved in the JA pathway (but not in SA, whose expression did not significantly change). The results from a first overview generally confirmed that the application of these beneficial fungi against one of the most economically important pathogens, the necrotrophic fungus *B. cinerea*, offers interesting perspectives, considering the polyphagous lifestyle of the pathogen as well as the global economic importance of the host plants [8]. *B. cinerea* is generally recognized as one of the most important pathogens affecting tomato also in terms of post-harvest quality, where alternatives to fungicides are under development in order to reduce the losses of fresh vegetables and fruits. Since the contribution of methyl jasmonate (MJ) in enhancing tomato resistance to *B. cinerea* and decreasing post-harvest losses in tomato has been recently reported [28], a deeper investigation into the effects of *Trichoderma* spp. on this pathway could improve the knowledge about the efficacy of these beneficial fungi also in post-harvest.

Among the six tomato species analyzed in the selected papers, based on the exclusion criteria here assessed for meta-regression, only in two (*S. lycorpesicum* and *S. habrochaites*) was it possible to perform the analysis, resulting in a different response of the host to the application of *Trichodema* spp. In particular, a higher disease severity decrease was observed for *S. habrochaites* if compared with *S. lycorpesicum*, with no significant difference in the expression of defense-related genes, although *TomloxA* and *TomloxC* were overexpressed only when *Trichoderma* spp. were inoculated on *S. habrochaites* and *S. lycorpesicum*, respectively. This varied response is not surprising since it is well known that the activation of plant signaling pathways and the reprogramming of plant gene expression by *Trichoderma* spp. strongly depend on the plant genotype [29,30]. Specifically, *S. lycorpesicum* is the only cultivated species included in the present meta-analysis, whereas *S. habrochaites* is just an important source of genetic variation for the crop improvement of *S. lycorpesicum*. However, it would be interesting to broaden this kind of analysis, it is not possible in the present work due to the availability of a single paper for each of the other four *Solanum* species reviewed here. 

With respect to *Trichoderma* spp., which was used in the selected articles, the meta-regression analysis did not highlight a significant difference based on the species these isolates belong to, i.e., *T. harzianum, T. asperellum, T. atroviride* and *T. virens*, since each one seemed to guarantee a significant level of disease control (disease severity), with *T. atroviride* and *T. virens* as the most efficient. However, any difference occurred when the expression of defense-related genes was compared among isolates, even if, due to the initial selection, the analysis was possible only in the case of *T. asperellum* and *T. harzianum* application and only on four genes connected with JA (*PINI*, *PINII*, *TomloxA*, *TomloxC*) and on two involved in SA (*PR1b1* and *PR-P2*) pathways. The four species previously listed are those including the higher number of isolates historically investigated as potential biocontrol agents [31] and used as active ingredients of commercial biopesticides [31,32]. However, it is important to consider that the *Trichoderma* taxonomy is continuously under revision and a new species description, as well as an update of what actually indicated as a species complex (see the case of *T. harzianum*), is still in progress [31,33,34]. The data reported here did not include the other species that are used in the selected papers (*T. koningiopsis*, *T. arundinaceum*, *T. longibrachiatum*, *T. parareseei*) because they did not fulfil the selection criteria established for the present work.

From a more practical point of view, the meta-regression analysis was performed considering the method of application and the duration of the treatment as indicators of the beneficial effect of *Trichoderma* spp. on tomato leaves in presence of *B. cinerea*. Among all the types of application reported in the selected papers, only seed and soil inoculations were comparable, since leaf treatment did not pass the chosen threshold. However, no difference in disease control neither in defense-related gene expression (*PINI*) was recorded between the two methods of application. Both seed and soil treatments were usually chosen as the preferred mode to inoculate beneficial *Trichoderma* isolates, including those formulated in commercial products, since they assure that mechanisms of action, such as mycoparasitism [35], competition for nutrients and space [36,37] and antagonism [38] vs. plant pathogenic organisms, as well as the endophytical colonization of the first layers of root cells, a prerequisite for the induction of defense response in the host [39], can be quickly and fully exerted. This last mechanism corresponds to what Vos and collaborators called the “*primed state of the plant*”, a unique physiological situation where the primed plants can respond more rapidly and/or more strongly when subsequently challenged by pathogens [8].

The duration of *Trichoderma* spp. treatment did not seem to affect disease control in terms of disease severity and *PINI* and *TomloxA* gene expression. Differently, *PINII* was more overexpressed between 21 and 35 days than after 35 days, while the overexpression of *PR1b1* and *PR-R2* genes can be registered only in the period 21–35 DOT. This could be in line with what is already known about the three-actor interactions, where *Trichoderma* spp. can generally activate both the SA- and JA/Et-mediated signal transduction pathways, but plant response can vary depending on the experimental conditions, as well as the organisms involved [40,41], as also confirmed by the variable responses obtained from this meta-analysis. 

Finally, the last three parameters (tomato phenological stage at *B. cinerea* or *Trichoderma* treatment and number days of *B. cinerea* infection) did not seem to affect disease severity, while information about the other parameters is not available. However, the phenological stage of both the pathogen and biocontrol agent, as well as the duration of pathogen infection, should be better analyzed in order to clarify the real efficacy of the beneficial isolate.

In conclusion, despite the relatively few comparisons made here, due to the lack of homogeneity in the parameters included in the selected works and to the stringent selection criteria adopted for the analysis, the present work can be considered as the first comprehensive investigation of the cross-talk occurring in the tri-partite system *Trichoderma*–tomato–*B. cinerea* using a meta-analysis approach. The outcomes of this study may contribute to a better understanding of the available literature concerning the modulation of the defense response in tomato against the pathogen by *Trichoderma* spp., and some weak points of these studies were also highlighted in order to improve the conceptualization and measure of future studies.

## 4. Materials and Methods

### 4.1. Database

A database reporting variations in *B. cinerea* infection and defense-related genes in tomato due to *Trichoderma* spp. treatment was created by examining the published peer-reviewed literature, searched in the Web of Science (Thompson-ISI, Philadelphia, PA, USA, http://apps.webofknowledge.com/, accessed on 1 December 2021) and Scopus (Elsevier, Amsterdam, The Netherlands, http://www.scopus.com/, accessed on 1 December 2021) databases, using “*Trichoderma*”, “*Botrytis cinerea*”, “tomato” and “induction of resistance” as keywords. Database searches were performed in August 2021 and spanned back to 2010. The reference lists of any article identified by this literature search were cross-checked in order to include any other relevant reference, finally identifying 40 research papers focused on any interactions among tomato, *B. cinerea* and/or *Trichoderma*. Articles and their data were excluded if (i) they were review articles, books or book chapters; (ii) they only included in vitro assays, such as antibiosis tests performed in plates; (iii) standard error (SE), standard deviation (SD) or replications were not reported; (iv) contained only commercial formulated isolates and lacked information about the amount of *Trichoderma* spp. used; (v) they did not use *Botrytis cinerea* treatment as control and/or *B. cinerea* + *Trichoderma* spp. as treatment. After removing articles that did not match these criteria, 15 research papers were included in the present meta-analysis (Appendix A). The articles were examined for parameters related to *B. cinerea* infection and defense-related genes. In order to compare the parameters related to *B. cinerea* infection, they were properly classified as “disease severity” (e.g., the area of plant tissue that is symptomatic), “disease incidence” (e.g., the number of diseased plants in a population), and “disease intensity” (taking into account both disease severity and incidence) [42], and if not already expressed as a percentage, they were converted to percentages (for this conversion, if the value equal to 100% was not determinable, the maximum mean+SD value was considered to be 100%). For each parameter observation, the mean values under control, i.e., *B. cinerea* infection without *Trichoderma* treatment, or treated conditions, i.e., *B. cinerea* infection with *Trichoderma* treatment ($\bar{X}c$ and $\bar{X}t$, respectively), as well as their standard deviations and number of replications, were directly obtained from table or text, if reported; otherwise, they were extrapolated from graphs using the GetData Graph Digitizer (v. 2.26; http://getdata-graph-digitizer.com/, accessed on 1 December 2021). All these values were associated in the database with the categorical information (see Section 4.2; Table S1), including the concentration of the *Trichoderma* suspensions used.

Since the methods for meta-analyses require that single observations are statistically independent, within each study, parameter values were recognized as independent if they were collected on different tomato species or varieties/cultivars/lines within a species, as well as on samples treated with different *Trichoderma* species or strains within a species, suspension concentrations, and for different durations [43,44]. Two of the selected articles, which tested three concentrations of *Thrichoderma* suspensions or four *Trichoderma* strains, presented non-independence issues due to shared controls among experimental groups (i.e., plants treated with *Trichoderma*); this issue was solved by splitting the shared controls into three or four groups, respectively, according to previous meta-analyses (e.g., Cotrozzi [45]).

### 4.2. Sources of Variation

The effect of *Trichoderma* on *B. cinerea* infection and defense-related genes in tomato was investigated for the following seven categories: (i) tomato species (*S. chilense*, *S. habrochaites*, *S. lycoprsicoides*, *S. lycopersicum*, *S. pennellii*, *S. pimpinellifolium*), (ii) tomato phenological stage at *B. cinerea* infection (seedling or mature plant if one month-older), (iii) *Trichoderma* species (*T. arundinaceum*, *T. asperellum*, *T. atroviride*, *T. harzianum*, *T. koningiopsis*, *T. longibrachiatum*, *T. parareseei*, *T. virens*), (iv) *Trichoderma* treatment type (seed, soil, leaf), (v) tomato phenological stage at *Trichoderma* treatment (sowing, seedling, mature plant), (vi) duration of *Trichoderma* treatment (four classes were determined: 0–5, 6–20, 21–35, and >35 days of treatment, DOT), and (vii) duration of *B. cinerea* infection (four classes were determined: 0–3, 4–7, 8–14, and >14 days of infection, DOI).

### 4.3. Meta-Analyses

To perform the meta-analyses, the software OpenMee (Brown University, Providence, RI, USA; [46]) was used, and the natural log of the response ratio (*r* = $\bar{X}c/\bar{X}t$) was used as the metric for estimating the effect of *Trichoderma*, according to Rosenberg et al. [47]. According to previous meta-analyses [43,44,48,49,50], effect sizes are reported as the *r* converted to the mean percentage change from the control as (*r* − 1) × 100. Negative percentage changes indicate a decrease in the parameter in response to *Trichoderma* treatment, while positive values indicate an increase. Based on the assumption of random variation in effect sizes between studies, a weighted mixed-model analysis was used, where each individual response was weighted by the reciprocal of the mixed-model variance [43,51,52]. Effect size estimates were considered significant when the 95% confidential intervals (CI) did not overlap zero [44].

For each category listed in the sources of variation, between-group heterogeneity (*Q_B_*) was assessed, and if *Q_B_* was significant (*p* ≤ 0.05), data were subdivided based on the levels of these categorical variables (i.e., meta-regression). Means were considered significantly different among them when there was no overlapping of the 95% CI [47].

Parameters and levels of each category listed in the source of variation were included in these analyses (i.e., meta-analysis and meta-regression, respectively) if there was a minimum of 6 observations; otherwise, results were only discussed if they originated from two or more independent papers, in order to make the results more robust, according to previous meta-analyses [43,44,48,49].

## Figures and Tables

**Table 1:** Between-group heterogeneity (*Q_B_*), degrees of freedom (*df*) and *p*-value (*p*) for the effect size of *Trichoderma* on *Botrytis cinerea* infection (i.e., disease intensity, severity and incidence) and defense-related genes involved in jasmonic (JA; i.e., proteinase inhibitors I and II, *PINI* and *PINII*, and tomato lipoxygenase A and C, *TomloxA* and *TomloxC*) and salycilic acid (SA; pathogenesis-related 1b1 and P2, *PR1b1* and *PR-P2*) pathways in tomato, across descriptive categories (tomato species, Tom sp; tomato phenological stage at *B. cinerea* infection, Tom PS *Bc* I; *Trichoderma* species, *Tricho* sp; *Trichoderma* treatment type, *Tricho* TT; tomato phenological stage at *Trichoderma* treatment, Tom PS *Tricho* T; duration of *Trichoderma* treatment, *Thricho* TD; duration of *B. cinerea* infection, *Bc* ID). Significant *p* are shown in bold. N.A.: not available (i.e., not included in meta-regression after exclusion criteria were assessed).

Parameter	Tom sp			Tom PS *Bc* I			*Tricho* sp			*Tricho* TT			Tom PS *Tricho* T			*Tricho* TD			*Bc* ID		
	*Q_B_*	*df*	*p*	*Q_B_*	*df*	*p*	*Q_B_*	*df*	*p*	*Q_B_*	*df*	*p*	*Q_B_*	*df*	*p*	*Q_B_*	*df*	*p*	*Q_B_*	*df*	*p*
Dis. Intensity	N.A.			N.A.			N.A.			N.A.			N.A.			N.A.			6.03	2	**0.049**
Dis. Severity	33.70	1	**<0.001**	0.58	1	0.447	19.50	3	**<0.001**	0.98	1	0.321	4.46	2	0.108	2.63	2	0.268	1.75	2	0.416
Dis. Incidence	N.A.			N.A.			N.A.			N.A.			N.A.			N.A.			N.A.		
*PINI* (JA)	0.13	1	0.719	N.A.			2.50	1	0.114	N.A.			N.A.			0.93	1	0.335	0.93	1	0.335
*PINII* (JA)	N.A.			N.A.			1.32	1	0.251	0.44	1	0.509	N.A.			7.29	1	**0.007**	8.34	1	**0.004**
*TomloxA* (JA)	14.10	1	**<0.001**	N.A.			1.16	1	0.282	N.A.			N.A.			2.17	1	0.140	2.24	1	0.135
*TomloxC* (JA)	14.60	1	**<0.001**	N.A.			0.12	1	0.730	N.A.			N.A.			N.A.			N.A.		
*PR1b1* (SA)	2.14	1	0.144	N.A.			0.02	1	0.875	N.A.			N.A.			23.70	1	**<0.001**	23.70	1	**<0.001**
*PR-P2* (SA)	0.57	1	0.452	N.A.			1.00	1	0.328	N.A.			N.A.			27.40	1	**<0.001**	27.40	1	**<0.001**

## Data Availability

All data included in the main text.

## Supplementary Materials

The following are available online at https://www.mdpi.com/article/10.3390/plants11020180/s1, Table S1. Database of the effects of *Trichoderma* on *Botrytis cinerea* infection and defense-related genes in tomato; Table S2. Details of meta-analysis on the effects of *Trichoderma* on *Botrytis cinerea* infection and defense-related genes involved in jasmonic and salicilic acid pathways in tomato; Table S3. Details of meta-regression on the effects of *Trichoderma* on *Botrytis cinerea* infection and defense-related genes in tomato; Tables S4–S10. Numbers of observations and studies within levels of descriptive categories.

## Appendix A. Articles Used in the Present Meta-Analysis on the Effects of *Trichoderma* on *Botrytis cinerea* Infection and Defense-Related Genes in Tomato

1. Yin, G.; Wang, W.; Sha, S.; Liu, L.; Yu, X. Inhibition and control effects of the ethyl acetate extract of *Trichoderma harzianum* fermented broth against *Botrytis cinerea*. *Afr. J. Microbiol. Res.* **2010**, *4*, 1647–1653.
2. Tucci, M.; Ruocco, M.; De Masi, L.; De Palma, M.; Lorito, M. The beneficial effect of *Trichoderma* spp. on tomato is modulated by the plant genotype. *Mol. Plant Pathol.* **2011**, *12*, 341–354.
3. Malmierca, M.G.; Cardoza, R.E.; Alexander, N.J.; McCormick, S.P.; Hermosa, R.; Monte, E.; Gutiérrez, S. Involvement of *Trichoderma* trichothecenes in the biocontrol activity and induction of plant defense-related genes. *Appl. Environ. Microbiol.* **2012**, *78*, 4856–4868.
4. Martínez-Medina, A.; Fernández, I.; Sánchez-Guzmán, M.J.; Jung, S.C.; Pascual, J.A.; Pozo, M.J. Deciphering the hormonal signalling network behind the systemic resistance induced by *Trichoderma harzianum* in tomato. *Front. Plant Sci.* **2013**, *4*, 206.
5. Cardoza, R.E.; Malmierca, M.G.; Gutiérrez, S. Overexpression of erg1 gene in *Trichoderma harzianum* CECT 2413: effect on the induction of tomato defence-related genes. *J. Appl. Microbiol.* **2014**, *117*, 812–823.
6. Fernández, E.; Segarra, G.; Trillas, M.I. Physiological effects of the induction of resistance by compost or *Trichoderma asperellum* strain T34 against *Botrytis cinerea* in tomato. *Biol. Control* **2014**, *78*, 77–85.
7. Harel, Y.M.; Mehari, Z.H.; Rav-David, D.; Elad, Y. Systemic resistance to gray mold induced in tomato by benzothiadiazole and *Trichoderma harzianum* T39. *Phytopathology* **2014**, *104*, 150–157.
8. Rubio, M.B.; Quijada, N.M.; Pérez, E.; Domínguez, S.; Monte, E.; Hermosa, R. Identifying beneficial qualities of *Trichoderma parareesei* for plants. *Appl. Environ. Microbiol.* **2014**, *80*, 1864–1873.
9. Salas-Marina, M.A.; Isordia-Jasso, M.I.; Islas-Osuna, M.A.; Delgado-Sánchez, P.; Jiménez-Bremont, J.F.; Rodríguez-Kessler, M.; Rosales-Saavedra, M.T.; Herrera-Estrella, A.; Casas-Flores, S. The Epl1 and Sm1 proteins from *Trichoderma atroviride* and *Trichoderma virens* differentially modulate systemic disease resistance against different life style pathogens in *Solanum lycopersicum*. *Front. Plant Sci.* **2015**, *6*, 77.
10. De Palma, M.; D’Agostino, N.; Proietti, S.; Bertini, L.; Lorito, M.; Ruocco, M.; Caruso, C.; Chiusano, M.L.; Tucci, M. Suppression subtractive hybridization analysis provides new insights into the tomato (*Solanum lycopersicum* L.) response to the plant probiotic microorganism *Trichoderma longibrachiatum* MK1. *J. Plant Physiol.* **2016**, *190*, 79–94.
11. You, J.; Zhang, J.; Wu, M.; Yang, L.; Chen, W.; Li, G. Multiple criteria-based screening of *Trichoderma* isolates for biological control of *Botrytis cinerea* on tomato. *Biol. Control* **2016**, *101*, 31–38.
12. Fernández, E.; Trillas, M.I.; Segarra, G. Increased rhizosphere populations of *Trichoderma asperellum* strain T34 caused by secretion pattern of root exudates in tomato plants inoculated with *Botrytis cinerea*. *Plant Pathol.* **2017**, *66*, 1110–1116.
13. Sarrocco, S.; Matarese, F.; Baroncelli, R.; Vannacci, G.; Seidl-Seiboth, V.; Kubicek, C.P.; Vergara, M. The constitutive endopolygalacturonase TvPG2 regulates the induction of plant systemic resistance by *Trichoderma virens*. *Phytopathology* **2017**, *107*, 537–544.
14. Herrera-Téllez, V.I.; Cruz-Olmedo, A.K.; Plasencia, J.; Gavilanes-Ruíz, M.; Arce-Cervantes, O.; Hernández-León, S.; Saucedo-García, M. The protective effect of *Trichoderma asperellum* on tomato plants against *Fusarium oxysporum* and *Botrytis cinerea* diseases involves inhibition of reactive oxygen species production. *Int. J. Mol. Sci.* **2019**, *20*, 2007.
15. Jaiswal, A.K.; Mengiste, T.D.; Myers, J.R.; Egel, D.S.; Hoagland, L.A. Tomato domestication attenuated responsiveness to a beneficial soil microbe for plant growth promotion and induction of systemic resistance to foliar pathogens. *Front. Microbiol.* **2020**, *11*, 3309.
