# Supplementary material for: Trichoderma-Induced Resistance to Botrytis cinerea in Solanum Species: A Meta-Analysis
Source: Plants (Basel). 2022 Jan 11;11(2):180. doi: 10.3390/plants11020180 (PMC8780288; doi:10.3390/plants11020180)
Supplement: Supplementary file 1 [file plants-11-00180-s001.zip › plants-1535873-supplementary.pdf]

Table S1. Database of the effects of *Trichoderma* on *Botrytis cinerea* infection and defense-related genes in tomato. Year, tomato species (Tom sp), tomato phenological stage at *B. cinerea* infection (Tom PS *Bc* I), *Trichoderma* species (*Tricho* sp), *Trichoderma* treatment type (*Tricho* TT) tomato phenological stage at *Trichoderma* treatment (Tom PS *Tricho* T), and duration of *Trichoderma* treatment (*Tricho* TD; days of treatment, DOT) and duration of *B. cinerea* infection (*Bc* ID; days of infection, DOI) used in the reviewed studies. See Appendix A for references. N.A.: not available. *: no standard error or standard deviation were reported.

Authors	Year	Tom sp	Tom PS *Bc* I	*Tricho* sp	*Tricho* TT	Tom PS *Tricho* T	*Tricho* TD (DOT)	*Bc* ID (DOI)
Yin et al.	2010	*S. lycopersicum*	Seedling	*T. harzianum*	Leaf	Seedling	6-20	4-7, 8-14, >14
Tucci et al.*	2011	*S. habrochaites*, *S. lycopersicum*	Mature plant	*T. atroviridae*, *T. harzianum*	Seed	Sowing	>35	0-3, 4-7
Malmierca et al.	2012	*S. lycopersicum*	Mature plant	*T. arundinaceum*	Seed	Sowing	21-35	4-7
Martìnez-Medina et al.	2013	*S. lycopersicum*	Mature plant	*T. harzianum*	Soil	Seedling	21-35	4-7
Cardoza et al.	2014	*S. lycopersicum*	Mature plant	*T. harzianum*	Seed	Sowing	21-35	4-7
Fernandez et al.	2014	*S. lycopersicum*	Seedling	*T. asperellum*	Soil	Seedling	0-5, 6-20	0-3, 4-7, 8-14
Harel et al.	2014	*S. lycopersicum*	Mature plant	*T. harzianum*	Soil	Mature plant	0-5, 6-20	0-3, 4-7
Rubio et al.	2014	*S. lycopersicum*	Mature plant	*T. parareseei*	Soil	Mature plant	6-20	4-7
Salas-Marina et al.	2015	*S. lycopersicum*	Seedling	*T. atroviridae*, *T. virens*	Soil	Seedling	21-35	8-14
De Palma et al.	2016	*S. lycopersicum*	Mature plant	*T. longibrachiatum*	Seed	Sowing	>35	0-3
You et al.	2016	*S. lycopersicum*	Seedling	*T. harzianum*, *T. koningiopsis*	Leaf	Seedling	6-20	4-7
Fernandez et al.	2017	*S. lycopersicum*	Seedling	*T. asperellum*	Soil	Seedling	6-20	4-7, 8-14
Sarrocco et al.	2017	*S. lycopersicum*	Seedling	*T. virens*	Soil	Seedling	21-35	8-14
Herrera-Téllez et al.	2019	*S. lycopersicum*	Mature plant	*T. asperellum*	Soil	Seedling	21-35	4-7, 8-14
Jaiswal et al.	2020	*S. chilense*, *S. habrochaites*, *S. lycopersicoides*, *S. lycopersicum*, *S. pennellii.* *S. pimpinellifolium*	Mature plant	*T. harzianum*	Soil	Seedling	21-35	N.A.

Table S2. Details of meta-analysis on the effects of *Trichoderma* on *Botrytis cinerea* infection (i.e., disease intensity, severity and incidence) and defense-related genes involved in jasmonic (JA; i.e., proteinase inhibitors I and II, *PINI* and *PINII*, and tomato lipoxygenase A and C, *TomloxA* and *TomloxC*) and salicilic acid (SA; pathogenesis-related 1b1 and P2, *PR1b1* and *PR-P2*) pathways in tomato. For each parameter, outcomes for model results (effect size, *r*; 95% confidence interval, CI; standard error, SE; and *P*-value) and heterogeneity (between-study variance, τ^2; degrees of freedom, *df*; heterogeneity, *Q*; *P*-value; and inconsistency, I^2) are shown.

**Parameter**	**Model results**				**Heterogeneity**				
	r	**95% CI**	**SE**	***P***	τ^2	***df***	***Q***	***P***	I^2
Dis. Intensity	-0.830	-0.973 – -0.687	0.073	<0.001	0.161	30	7.87E+3	<0.001	99.6
Dis. Severity	-0.499	-0.582 – -0.416	0.042	<0.001	0.129	73	1.24E+11	<0.001	100.0
Disease Incidence	-0.047	-0.072 – -0.022	0.013	<0.001	0.000	6	6.52E+1	<0.001	90.8
PINI (JA)	0.329	0.043 – 0.614	0.146	0.024	0.680	31	8.06E+8	<0.001	100.0
PINII (JA)	0.846	0.400 – 1.292	0.228	<0.001	1.606	31	2.25E+10	<0.001	100.0
TomloxA (JA)	0.509	0.396 – 0.622	0.058	<0.001	0.114	33	2.41E+9	<0.001	100.0
TomloxC (JA)	0.406	0.243 – 0.569	0.083	<0.001	0.221	31	5.18E+7	<0.001	100.0
PR1b1 (SA)	0.318	-0.184 – 0.820	0.256	0.214	2.162	32	1.20E+9	<0.001	100.0
PR-P2 (SA)	0.320	-0.254 – 0.894	0.293	0.275	2.744	31	1.40E+9	<0.001	100.0

Table S3. Details of meta-regression on the effects of *Trichoderma* on *Botrytis cinerea* infection and defense-related genes in tomato. Number of observations (Obs), effect size (*r*), 95% confidence interval (CI), standard error (SE) and *P*-value are shown for each level of tomato species (Tom sp), *Trichoderma* species (*Tricho* sp), duration of *Trichoderma* treatment (*Tricho* TD; days of treatment, DOT) and duration of *B. cinerea* infection (*Bc* ID; days of infection, DOI) descriptive categories. For parameter abbreviations, see caption of Table S2.

Category	Level	Parameter	Obs	*r*	95% CI	SE	*P*
Tom sp	*S. habrochaites*	Disease Severity	4	-2.506	-2.536 – -2.475	0.016	<0.001
	S. lycopersicum	Disease Severity	64	-0.328	-0.417 – -0.239	0.045	<0.001
	S. habrochaites	*TomloxA* (JA)	6	1.672	1.435 – 1.908	0.121	<0.001
	S. lycopersicum	*TomloxA* (JA)	28	0.260	-0.108 – 0.629	0.188	0.166
	S. habrochaites	*TomloxC* (JA)	6	-0.240	-0.583 – 0.103	0.175	0.170
	S. lycopersicum	*TomloxC* (JA)	26	0.550	0.384 – 0.727	0.088	<0.001
Tricho sp	*T. asperellum*	Disease Severity	8	-0.388	-0.517 – -0.260	0.066	<0.001
	T. atroviridae	Disease Severity	11	-0.885	-1.127 – -0.643	0.123	<0.001
	T. harzianum	Disease. Severity	52	-0.353	-0.420 – -0.287	0.034	<0.001
	T. virens	Disease Severity	2	-4.533	-7.743 – -1.324	1.637	0.006
Tricho TD	21-35 DOT	*PINII* (JA)	6	2.739	2.044 – 3.434	0.355	<0.001
	>35 DOT	*PINII* (JA)	24	0.596	0.089 – 1.103	0.259	0.021
	21-35 DOT	*PR1b1* (SA)	2	3.252	1.453 – 5.502	0.918	<0.001
	>35 DOT	*PR1b1* (SA)	31	0.129	-0.146 – 0.404	0.140	0.357
	21-35 DOT	*PR-P2* (SA)	2	3.270	1.324 – 5.215	0.993	<0.001
	>35 DOT	*PR-P2* (SA)	31	0.123	-0.248 – 0.494	0.189	0.516
Bc ID	4-7 DOI	Disease Intensity	14	-0.955	-1.198 – -0.712	0.124	<0.001
	8-14 DOI	Disease Intensity	9	-0.760	-0.880 – -0.632	0.065	<0.001
	>14 DOI	Disease Intensity	8	-0.699	-0.849 – -0.548	0.077	<0.001
	0-3 DOI	*PINII* (JA)	26	0.492	0.005 – 0.979	0.249	0.048
	4-7 DOI	*PINII* (JA)	6	2.739	2.044 – 3.434	0.355	<0.001
	0-3 DOI	*PR1b1* (SA)	31	0.129	-0.146 – 0.404	0.140	0.357
	4-7 DOI	*PR1b1* (SA)	2	3.252	1.453 – 5.052	0.918	<0.001
	0-3 DOI	*PR-P2* (SA)	30	0.123	-0.248 – 0.494	0.189	0.516
	4-7 DOI	*PR-P2* (SA)	2	3.270	1.324 – 5.215	0.993	<0.001

Table S4. Numbers of observations (O) and studies (S) within levels of tomato species (*S. chilense, S. habrochaites, S. lycopersicoides, S. lycopersicum, S. pennellii, S. pimpinellifolium*) descriptive category. Parameters completely excluded from meta-regression are highlighted in yellow, whereas levels excluded from this analysis are reported in red. For parameter abbreviations, see caption of Table S2.

Parameter	All		Tomato species											
			S. chilense		*S. habrochaites*		*S. lycopersicoides*		*S. lycopersicum*		*S. pennellii*		*S. pimpinellifolium*	
	O	S	O	S	O	S	O	S	O	S	O	S	O	S
Disease Intensity	31	4	0	0	0	0	0	0	31	4	0	0	0	0
Disease Severity	74	11	1	1	4	2	1	1	64	10	2	1	2	1
Disease Incidence	7	2	0	0	0	0	0	0	7	2	0	0	0	0
PINI (JA)	32	3	0	0	6	1	0	0	26	3	0	0	0	0
PINII (JA)	32	5	0	0	0	0	0	0	32	5	0	0	0	0
TomloxA (JA)	34	4	0	0	6	1	0	0	28	4	0	0	0	0
TomloxC (JA)	32	2	0	0	6	1	0	0	26	2	0	0	0	0
PR1b1 (SA)	33	4	0	0	6	1	0	0	27	4	0	0	0	0
PR-P2 (SA)	32	3	0	0	6	1	0	0	26	3	0	0	0	0

Table S5. Numbers of observations (O) and studies (S) within levels of tomato phenological stage at *Botrytis cinerea* infection (seedling, mature plant) descriptive category. Parameters completely excluded from meta-regression are highlighted in yellow, whereas levels excluded from this analysis are reported in red. For parameter abbreviations, see caption of Table S2.

**Parameter**	**All**		**Tomato phenological stage at *Botrytis cinerea* infection**			
			**Seedling**		**Mature plant**	
	**O**	**S**	**O**	**S**	**O**	**S**
Disease Intensity	31	4	29	3	2	1
Disease Severity	74	11	46	7	9	2
Disease Incidence	7	2	7	2	0	0
PINI (JA)	32	3	0	0	0	0
PINII (JA)	32	5	4	1	2	1
TomloxA (JA)	34	4	0	0	2	1
TomloxC (JA)	32	2	0	0	2	1
PR1b1 (SA)	33	4	0	0	0	0
PR-P2 (SA)	32	3	0	0	0	0

Table S6. Numbers of observations (O) and studies (S) within levels of *Trichoderma* species (*T. arundinaceum, T. asperellum, T. atroviride, T. harzianum, T. koningiopsis, T. longibrachiatum, T. parareseei, T. virens*) descriptive categoriy. Parameters completely excluded from meta-regression are highlighted in yellow, whereas levels excluded from this analysis are reported in red. For parameter abbreviations, see caption of Table S2.

**Parameter**	**All**		***Trichoderma* species**															
			T. arundinaceum		***T. asperellum***		***T. atroviride***		***T. harzianum***		***T. koningiopsis***		***T. longibrachiatum***		***T. parareseei***		***T. virens***	
	**O**	**S**	**O**	**S**	**O**	**S**	**O**	**S**	**O**	**S**	**O**	**S**	**O**	**S**	**O**	**S**	**O**	**S**
Disease Intensity	31	4	0	0	1	1	0	0	28	3	2	1	0	0	0	0	0	0
Disease Severity	74	11	0	0	8	3	11	2	52	4	0	0	0	0	1	1	2	2
Disease Incidence	7	2	0	0	3	1	0	0	2	1	2	1	0	0	0	0	0	0
PINI (JA)	32	3	1	1	0	0	15	1	16	2	0	0	0	0	0	0	0	0
PINII (JA)	32	5	1	1	0	0	12	1	19	4	0	0	0	0	0	0	0	0
TomloxA (JA)	34	4	1	1	0	0	15	1	18	3	0	0	0	0	0	0	0	0
TomloxC (JA)	32	2	0	0	0	0	15	1	17	2	0	0	0	0	0	0	0	0
PR1b1 (SA)	33	4	1	1	0	0	15	1	16	2	0	0	1	1	0	0	0	0
PR-P2 (SA)	32	3	1	1	0	0	15	1	16	2	0	0	0	0	0	0	0	0

Table S7. Numbers of observations (O) and studies (S) within levels of *Trichoderma* treatment type (seed, soil, leaf) descriptive category. Parameters completely excluded from meta-regression are highlighted in yellow, whereas levels excluded from this analysis are reported in red. For parameter abbreviations, see caption of Table S2.

**Parameter**	**All**		***Trichoderma* treatment type**					
			**Seed**		**Soil**		**Leaf**	
	**O**	**S**	**O**	**S**	**O**	**S**	**O**	**S**
Disease Intensity	31	4	0	0	3	1	28	2
Disease Severity	74	11	19	1	55	9	0	0
Disease Incidence	7	2	0	0	3	1	4	1
PINI (JA)	32	3	32	3	0	0	0	0
PINII (JA)	32	5	26	3	6	2	0	0
TomloxA (JA)	34	4	32	3	2	1	0	0
TomloxC (JA)	32	2	30	1	2	1	0	0
PR1b1 (SA)	33	4	33	4	0	0	0	0
PR-P2 (SA)	32	3	32	3	0	0	0	0

Table S8. Numbers of observations (O) and studies (S) within levels of tomato phenological stage at *Trichoderma* treatment (sowing, seedling, mature plant) descriptive category. Parameters completely excluded from meta-regression are highlighted in yellow, whereas levels excluded from this analysis are reported in red. For parameter abbreviations, see caption of Table S2.

Parameter	All		Tomato phenological stage at *Trichoderma* treatment					
			Sowing		Seedling		Mature plant	
	O	S	O	S	O	S	O	S
Disease Intensity	31	4	0	0	29	3	2	1
Disease Severity	74	11	19	1	46	7	9	2
Disease Incidence	7	2	0	0	7	2	0	0
PINI (JA)	32	3	32	3	0	0	0	0
PINII (JA)	32	5	26	3	4	1	2	1
TomloxA (JA)	34	4	32	3	0	0	2	1
TomloxC (JA)	32	2	30	1	0	0	2	1
PR1b1 (SA)	33	4	33	4	0	0	0	0
PR-P2 (SA)	32	3	32	3	0	0	0	0

Table S9. Numbers of observations (O) and studies (S) within levels of duration of *Trichoderma* treatment (0-5, 6-20, 21-35, >35 days of treatment, DOT) descriptive category. Parameters completely excluded from meta-regression are highlighted in yellow, whereas levels excluded from this analysis are reported in red. For parameter abbreviations, see caption of Table S2.

**Parameter**	**All**		**Duration of *Trichoderma* treatment**							
			**0-5 DOT**		**6-20 DOT**		**21-35 DOT**		**>35 DOT**	
	**O**	**S**	**O**	**S**	**O**	**S**	**O**	**S**	**O**	**S**
Disease Intensity	31	4	0	0	31	4	0	0	0	0
Disease Severity	74	11	0	0	15	4	40	5	19	1
Disease Incidence	7	2	0	0	7	2	0	0	0	0
PINI (JA)	32	3	0	0	0	0	2	2	30	1
PINII (JA)	32	5	2	1	0	0	6	3	24	1
TomloxA (JA)	34	4	2	1	0	0	2	2	30	1
TomloxC (JA)	32	2	2	1	0	0	0	0	30	1
PR1b1 (SA)	33	4	0	0	0	0	2	2	31	1
PR-P2 (SA)	32	3	0	0	0	0	2	2	30	1

Table S10. Numbers of observations (O) and studies (S) within levels of duration of *Botrytis cinerea* infection (0-3, 4-7, 8-14, >14 days of infection, DOI) descriptive category. Parameters completely excluded from meta-regression are highlighted in yellow, whereas levels excluded from this analysis are reported in red. N.A.: not available (thus excluded from meta-regression). For parameter abbreviations, see caption of Table S2.

**Parameter**	**All**		**Duration of *Botrytis cinerea* infection**									
			**0-3 DOI**		**4-7 DOI**		**8-14 DOI**		**>14 DOI**		**N.A.**	
	**O**	**S**	**O**	**S**	**O**	**S**	**O**	**S**	**O**	**S**	**O**	**S**
Disease Intensity	31	4	0	0	14	3	9	2	8	1	0	0
Disease Severity	74	11	11	2	30	7	8	5	0	0	25	1
Disease Incidence	7	2	0	0	5	2	2	1	0	0	0	0
PINI (JA)	32	3	30	1	2	2	0	0	0	0	0	0
PINII (JA)	32	5	26	2	6	3	0	0	0	0	0	0
TomloxA (JA)	34	4	32	2	2	2	0	0	0	0	0	0
TomloxC (JA)	32	2	32	2	0	0	0	0	0	0	0	0
PR1b1 (SA)	33	4	31	2	2	2	0	0	0	0	0	0
PR-P2 (SA)	32	3	30	1	2	2	0	0	0	0	0	0
